# Coping with COVID: risk and resilience factors for mental health in a German representative panel study

**DOI:** 10.1017/S0033291722000563

**Published:** 2022-03-01

**Authors:** Antje Riepenhausen, Ilya M. Veer, Carolin Wackerhagen, Zala C. Reppmann, Göran Köber, José Luis Ayuso-Mateos, Sophie A. Bögemann, Giovanni Corrao, Mireia Felez-Nobrega, Josep Maria Haro Abad, Erno Hermans, Judith van Leeuwen, Klaus Lieb, Vincent Lorant, Murielle Mary-Krause, Roberto Mediavilla, Maria Melchior, Ellenor Mittendorfer-Rutz, Matteo Monzio Compagnoni, Kuan-Yu Pan, Lara Puhlmann, Karin Roelofs, Marit Sijbrandij, Pierre Smith, Oliver Tüscher, Anke Witteveen, Matthias Zerban, Raffael Kalisch, Hannes Kröger, Henrik Walter

**Affiliations:** 1 Department of Psychiatry and Neurosciences - CCM, Research Division of Mind and Brain, Charité – Universitätsmedizin Berlin, Corporate Member of Freie Universität Berlin and Humboldt-Universität zu Berlin, Charitéplatz 1, 10117 Berlin, Germany; 2 Berlin School of Mind and Brain, Faculty of Philosophy, Humboldt-Universität zu Berlin, Berlin, Germany; 3 Department of Developmental Psychology, University of Amsterdam, Amsterdam, The Netherlands; 4 Institute of Medical Biometry and Statistics, Faculty of Medicine and Medical Center, University of Freiburg, Freiburg, Germany; 5 Freiburg Center for Data Analysis and Modelling, University of Freiburg, Freiburg, Germany; 6 Department of Psychiatry, Universidad Autónoma de Madrid (UAM), Madrid, Spain; 7 Centro de Investigación Biomédica en Red de Salud Mental (CIBERSAM), Madrid, Spain; 8 Department of Psychiatry, Instituto de Investigación Sanitaria Princesa (IIS-Princesa), La Princesa University Hospital, Madrid, Spain; 9 Donders Institute for Brain, Cognition, and Behaviour, Radboud University Medical Center, Nijmegen, The Netherlands; 10 Unit of Biostatistics, Epidemiology and Public Health, Department of Statistics and Quantitative Methods, University of Milano-Bicocca, Milan, Italy; 11 National Centre for Healthcare Research and Pharmacoepidemiology, University of Milano-Bicocca, Milan, Italy; 12 Research and Development Unit, Parc Sanitari Sant Joan de Déu, Barcelona, Spain; 13 Leibniz Institute for Resilience Research (LIR), Mainz, Germany; 14 Department of Psychiatry and Psychotherapy, University Medical Center Mainz, Mainz, Germany; 15 Institute of Health and Society (IRSS), Université Catholique de Louvain, Brussels, Belgium; 16 Department of Social Epidemiology, Institut Pierre Louis d'Epidémiologie et de Santé Publique, Sorbonne Université, INSERM, 75012 Paris, France; 17 Department of Clinical Neuroscience, Division of Insurance Medicine, Karolinska Institutet, Berzelius väg 3, 17177 Stockholm, Sweden; 18 Department of Psychiatry, Amsterdam Public Health, Amsterdam University Medical Center, Vrije Universiteit, Amsterdam, The Netherlands; 19 Research Group Social Stress and Family Health, Max Planck Institute for Human Cognitive and Brain Sciences, Leipzig, Germany; 20 Behavioural Science Institute, Radboud University Nijmegen, Nijmegen, The Netherlands; 21 Department of Clinical, Neuro- and Developmental Psychology, Amsterdam Public Health Research Institute and WHO Collaborating Center for Research and Dissemination of Psychological Interventions, Vrije Universiteit, Amsterdam, The Netherlands; 22 Department Epidemiology and Public Health, Sciensano, Brussels, Belgium; 23 Neuroimaging Center (NIC), Focus Program Translational Neuroscience (FTN), Johannes Gutenberg University Medical Center, Mainz, Germany; 24 Socio-Economic Panel (SOEP), German Institute for Economic Research (DIW), Berlin, Germany; 25 Munich Center for the Economics of Aging (MEA), Max Planck Institute for Social Law and Social Policy, Munich, Germany

**Keywords:** COVID-19, mental health, psychological distress, resilience, risk factors

## Abstract

**Background:**

The coronavirus disease 2019 (COVID-19) pandemic might affect mental health. Data from population-representative panel surveys with multiple waves including pre-COVID data investigating risk and protective factors are still rare.

**Methods:**

In a stratified random sample of the German household population (*n* = 6684), we conducted survey-weighted multiple linear regressions to determine the association of various psychological risk and protective factors assessed between 2015 and 2020 with changes in psychological distress [(PD; measured via Patient Health Questionnaire for Depression and Anxiety (PHQ-4)] from pre-pandemic (average of 2016 and 2019) to peri-pandemic (both 2020 and 2021) time points. Control analyses on PD change between two pre-pandemic time points (2016 and 2019) were conducted. Regularized regressions were computed to inform on which factors were statistically most influential in the multicollinear setting.

**Results:**

PHQ-4 scores in 2020 (*M* = 2.45) and 2021 (*M* = 2.21) were elevated compared to 2019 (*M* = 1.79). Several risk factors (catastrophizing, neuroticism, and asking for instrumental support) and protective factors (perceived stress recovery, positive reappraisal, and optimism) were identified for the peri-pandemic outcomes. Control analyses revealed that in pre-pandemic times, neuroticism and optimism were predominantly related to PD changes. Regularized regression mostly confirmed the results and highlighted perceived stress recovery as most consistent influential protective factor across peri-pandemic outcomes.

**Conclusions:**

We identified several psychological risk and protective factors related to PD outcomes during the COVID-19 pandemic. A comparison of pre-pandemic data stresses the relevance of longitudinal assessments to potentially reconcile contradictory findings. Implications and suggestions for targeted prevention and intervention programs during highly stressful times such as pandemics are discussed.

The spread of the severe acute respiratory syndrome coronavirus 2 (SARS-CoV-2) globally affects people in various aspects of their life. Not only does the virus impose a physical threat of infection and the associated possibility of a severe course with its long-term consequences; being exposed to such threat constantly, as well as to changes in social life and the economic situation can harm mental well-being. Indeed, several studies have investigated mental health consequences of the coronavirus disease 2019 (COVID-19) pandemic in nationally representative probability samples, most of them referring to the first lockdown in spring 2020 (see online Supplementary Table S1). With some exceptions, most of these studies found higher average levels of self-reported depression and anxiety symptoms during the first months of the pandemic compared to pre-pandemic symptom levels (Daly, Sutin, & Robinson, 2021; Dawel et al., 2020; Ettman et al., 2020; Peters, Rospleszcz, Greiser, Dallavalle, & Berger, 2020; Pieh, Budimir, & Probst, 2020; Pierce et al., 2020; Sibley et al., 2020; Twenge & Joiner, 2020; Winkler et al., 2020). Meta-analytic evidence from not exclusively representative studies suggests that these increases in psychological distress (PD) were relatively small and recovered over time (Prati & Mancini, 2021; Robinson, Sutin, Daly, & Jones, 2021).

Previous research on mental health during the COVID-19 pandemic has moreover identified several relevant demographic and socio-economic risk and protective factors. Higher PD during the COVID-19 pandemic has been consistently found to be associated with female gender (Daly & Robinson, 2020; Daly, Sutin, & Robinson, 2020; Gijzen et al., 2020; Holingue et al., 2020; Hyland et al., 2020; Li & Wang, 2020; Niedzwiedz et al., 2020; Peters et al., 2020; Pieh et al., 2020; Pierce et al., 2020; Zajacova et al., 2020), younger age (Daly & Robinson, 2020; Daly et al., 2020, 2021; Every-Palmer et al., 2020; Holingue et al., 2020; Hyland et al., 2020; Li & Wang, 2020; Niedzwiedz et al., 2020; Peters et al., 2020; Pieh et al., 2020; Pierce et al., 2020; Zajacova et al., 2020), pre-existing mental conditions (Daly & Robinson, 2020; Every-Palmer et al., 2020; Holman, Thompson, Garfin, & Silver, 2020), poor physical health status (Every-Palmer et al., 2020; Holman et al., 2020), and living with young children (Pierce et al., 2020). The results for level of education, income, and employment status are more heterogenous, with studies finding evidence of these being both potential protective as well as risk factors (Daly & Robinson, 2020; Daly et al., 2020; Ettman et al., 2020; Li & Wang, 2020; Niedzwiedz et al., 2020; Pieh et al., 2020; Pierce et al., 2020).

The impact of psychological factors on mental health during the COVID-19 pandemic, however, has received less attention particularly in representative studies. Identifying such – possibly malleable – psychological factors in the general population will be of great value for informing tailored prevention and intervention efforts to reduce mental health problems and improve well-being during crises (Kunzler et al., 2021).

Insights from studies using non-random convenience sampling suggest that several psychological factors are protective factors associated with lower PD or resilience (operationalized as lower PD than expected given a certain exposure to stressors) during the COVID-19 pandemic: these studies found lower PD to be predicted by cognitive flexibility (Dawson & Golijani-Moghaddam, 2020; McCracken, Badinlou, Buhrman, & Brocki, 2020), grit (McCracken et al., 2020), meaning in life (Schnell & Krampe, 2020), dispositional mindfulness (Conversano et al., 2020), secure and avoidant attachment styles (Moccia et al., 2020), optimism (Płomecka et al., 2020; Veer et al., 2021), emotional stability (i.e. low neuroticism; Fernández, Crivelli, Guimet, Allegri, & Pedreira, 2020; Flesia et al., 2020; Veer et al., 2021), self-control (Flesia et al., 2020; Schnell & Krampe, 2020), perceived stress recovery (Veer et al., 2021), positive appraisal style and positive appraisal specific to the COVID-19 pandemic (Veer et al., 2021), both positive (Flesia et al., 2020; Zhu et al., 2020) and behavioral (Veer et al., 2021) coping skills as well as coping skills specific for the COVID-19 pandemic (Fernández et al., 2020), making meaning in negative experiences (Yang et al., 2021), general self-efficacy (Bendau et al., 2020; Veer et al., 2021), internal locus of control (Flesia et al., 2020), and self-esteem (Arima et al., 2020).

Due to the non-random sampling strategy of most of the studies until now, it is however difficult to assess to what degree these results generalize to the general population or to what degree they might be driven by (self-) selection of the respondents into the sample (Fink, 2003). A further problem that prohibits reliable conclusions from previous studies on psychological factors and mental health during the COVID-19 pandemic is the systematic lack of pre-pandemic baseline measurements. Although these studies can thus describe PD during the pandemic or make claims on average changes by referring to average pre-pandemic health in other samples, they cannot draw inferences regarding measures of intra-individual change.

In the current study, we addressed both shortcomings in the literature and investigated the relationship between psychological factors (selected based on cross-sectional findings in a large convenience sample; Veer et al., 2021) and changes in depression and anxiety symptoms (PD) during the COVID-19 pandemic in a sample that is both representative of the German household population and has pre-pandemic baseline measures of the same individuals. Moreover, the long-running panel study allowed us to compare these associations to those with changes from 2016 to 2019, a ‘normal’ period without a singular and ubiquitous stressor like the pandemic.

In accordance with most studies on depression and anxiety in the pandemic, we assumed that the pandemic influenced PD, expecting an increase in PD in 2020 and 2021 compared to 2019 and 2016. We moreover hypothesized neuroticism and catastrophizing to be risk factors, expecting higher scores to be associated with larger increases or smaller decreases in PD. We finally expected the following psychological factors to be associated with smaller increases or larger decreases in PD, as protective factors: positive reappraisal, putting into perspective, acceptance, use of instrumental support, positive appraisal specific to the COVID-19 pandemic, perceived stress recovery, optimism, and locus of control.

## Methods

### Participants

The present sample is a subset of the German nationally representative panel study ‘Socio-economic Panel’ (SOEP; Goebel et al., 2019; Liebig et al., 2019). The SOEP annually surveys over 30 000 participants in more than 20 000 households which come from a stratified random sample of the German household population. For the current study, a random subset of 12 000 households (one participant per household) were contacted via telephone interviews between 1 April 2020 (67 366 confirmed cases of COVID-19 and 732 confirmed deaths related to COVID-19 in Germany so far) and 4 July 2020 (196 096 confirmed cases and 9010 confirmed deaths) in the context of the SOEP-CoV study (Entringer et al., 2020; Kühne, Kroh, Liebig, & Zinn, 2020). Data collection was split into nine tranches. Overall, *n* = 6684 individuals participated in the survey in 2020. All *n* = 6684 participants were recontacted between 18 January 2021 (2 040 659 confirmed cases and 46 633 confirmed deaths) and 15 February 2021 (2 338 987 confirmed cases and 65 076 confirmed deaths). Altogether, *n* = 6006 individuals participated in this follow-up survey. Participants were also surveyed in previous years and provided information on PD in 2016 (January–September; *N* = 5127) and 2019 (January–September; *N* = 6399); missing values for pre-pandemic PD were imputed. Information on exact timing and size of the individual tranches in 2020 and follow-up assessment in 2021 can be found in online Supplementary Table S2.

### Measures

PD was assessed using the Patient Health Questionnaire for Depression and Anxiety (PHQ-4), a four-item questionnaire screening for depressive and anxiety symptoms that has already been used in pre-pandemic waves in this sample (Kroenke, Spitzer, Williams, & Löwe, 2009; Löwe et al., 2010). The PHQ-4 is a validated mental health screening instrument and measures general anxiety and depressive symptoms using a 4-point Likert scale ranging from 0 (‘not at all’) to 3 (‘nearly every day’). Overall, sum scores range from 0 to 12 with classifications into no (0–2), mild (3–5), moderate (6–8), and severe (9–12) symptoms of general anxiety and depression. The PHQ-4 was assessed in 2016, 2019, 2020, and 2021.

The coping dimensions of positive reappraisal, putting into perspective and acceptance were assessed using three single items from the Cognitive Emotion Regulation Questionnaire (CERQ; Garnefski & Kraaij, 2007; Loch, Hiller, & Witthöft, 2011), adapted in wording to assess emotion regulation during the previous 2 weeks. Similarly, catastrophizing was measured using a reformulated item from the CERQ scale ‘catastrophizing’. The rationale for reformulating the CERQ items to reflect state- rather than trait-like coping was to capture emotion regulation strategies specifically used during the COVID-19 pandemic. Finally, instrumental support-seeking was measured using the first item of the ‘using instrumental support’ scale of the brief COPE (Carver, 1997). These coping items were selected because they were identified to load most strongly on three factors that were identified using principal component analysis in yet unpublished research (for details, see online Supplement S2). The items for positive reappraisal, putting into perspective and acceptance loaded most strongly on a factor representing positive appraisal style, using instrumental support best reflected a behavioral coping style factor, whereas catastrophizing best represented maladaptive coping.

Additionally, positive appraisal specific to the COVID-19 pandemic was assessed with two self-formulated items. Perceived stress recovery was measured using one item from the Brief Resilience Scale (Chmitorz et al., 2018; Smith et al., 2008). All coping, COVID-19 appraisal, and recovery items were answered on a Likert scale from 0 (‘don't agree at all’) to 4 (‘fully agree’) and were collected during the 2020 survey period. Optimism was assessed in 2019 using one item asking about the attitude toward the future, ranging from 1 (‘pessimistic’) to 4 (‘optimistic’). Locus of control was assessed in 2015 and measured using a 10-item instrument with a Likert scale ranging from 1 (‘disagree completely’) to 4 (‘agree completely’). Higher values indicate an internal locus of control. Neuroticism was assessed in 2017 using the Big Five Inventory – short version (BFI-S; Hahn, Gottschling, & Spinath, 2012). Answers on the 7-point Likert scale range from 1 (‘does not apply’) to 7 (‘applies fully’).

**Fig. 1.**
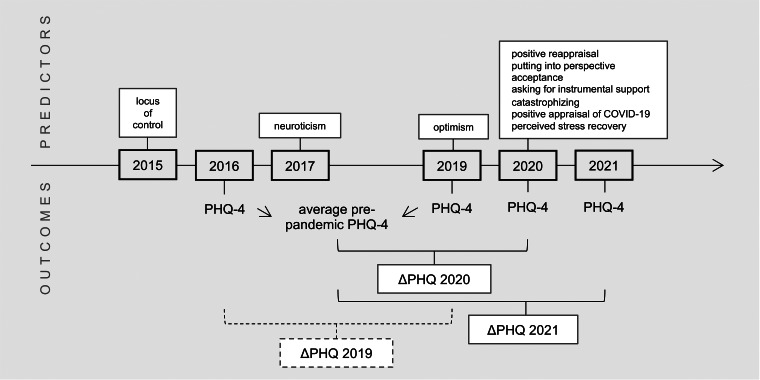
Timing of data collection for predictors and outcome variables. PHQ-4, Patient Health Questionnaire, 4 item version; ΔPHQ 2019, change in PHQ-4 from 2016 to 2019; ΔPHQ 2020, change in PHQ-4 from 2019 to 2020, ΔPHQ 2021, change in PHQ-4 from 2019 to 2021.

Example items for all measures and information on included covariates can be found in Table 1, which additionally summarizes the hypothesized relation between psychological factors and outcomes. An overview of the timing of data assessment for the different variables can be found in Fig. 1.

**Table 1.** Overview of variables and instruments used

Variable	Instrument	Type	Expected relation to PD
Dependent variables			
Change in PD from pre-pandemic levels (mean of 2016 and 2019) to 2020	ΔPHQ 2020 = PHQ-4 2020 − (PHQ-4 2016 + PHQ-4 2019)/2		
Change in PD from pre-pandemic levels (mean of 2016 and 2019) to 2021	ΔPHQ 2021 = PHQ-4 2021 − (PHQ-4 2016 + PHQ-4 2019)/2		
Change in PD from 2016 to 2019	ΔPHQ 2019 = PHQ-4 2019 − PHQ-4 2016		
Independent variables			
Coping: positive reappraisal	CERQ positive reappraisal scale (1 item); ‘I thought that the situation also has its positive sides’	State	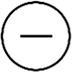
Coping: putting into perspective	CERQ putting into perspective scale (1 item); ‘I thought that it hasn't been too bad compared to other things’	State	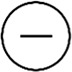
Coping: acceptance	CERQ acceptance scale (1 item); ‘I thought that I have to accept the situation’	State	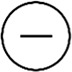
Positive appraisal specific to the COVID-19 pandemic	Self-formulated; positive appraisal of COVID-19 situation on a personal & societal level (2 items); ‘I expect that I will learn something positive from the corona pandemic for my own life’ and ‘In the long run, I think that society will change for the better because of the corona pandemic’	State	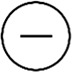
Coping: using instrumental support	Brief COPE: using instrumental support scale (1 item); ‘I've been trying to get advice or help from other people about what to do’	State	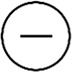
Coping: catastrophizing	CERQ: catastrophizing scale (1 item); ‘I kept thinking about how terrible it is what I have experienced’	State	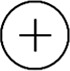
Perceived stress recovery	Brief Resilience Scale (1 item);	Trait	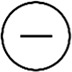
Optimism	SOEP-specific item (1 item); ‘If you think about the future, are you…’ (1, pessimistic – 4, optimistic)	Trait	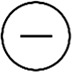
Locus of Control	SOEP-specific questionnaire (10 items); e.g. ‘My life's course depends on me’ and ‘Success is a matter of fate and luck’ (−)	Trait	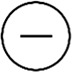
Neuroticism	BFI-S (3 items); ‘I am…’ ‘nervous’ ‘a worrier’, ‘relaxed, able to deal with stress’ (−)	Trait	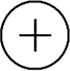
Covariates			
Age (in years)	18–24, 25–34, 35–44, 45–54, 55–64, 65–74, 75–84, 85+		
Gender	m/f		
Education	1: no degree, still in school or lower degree; 2: middle or high school degree; 3: high school degree with subsequent vocational training or university degree		
Household income	Lower/middle/upper tertile		
Risk group status for severe course in case of infection with SARS-CoV-2	Yes/no (determined based on age and BMI as well as self-report of at least one of the following diagnoses: asthma, cardiovascular disease, diabetes, cancer, stroke, high blood pressure, dementia, rheumatism, handicap)		
History of diagnosed depression	Yes/no (self-report)		
Lockdown status	Yes/no (participation in 2020 up to/after 5 May)		

PD, psychological distress; PHQ, Patient Health Questionnaire; CERQ, Cognitive Emotion Regulation Questionnaire; SOEP, Socio-economic Panel; BFI-S, Big Five Inventory, short version; m, male, f, female; SARS-CoV-2, severe acute respiratory syndrome coronavirus 2; BMI, body mass index.

*Note*. Expected relation to PHQ indicates the hypothesized relationship between the respective independent variable and ΔPHQ 2020 as well as ΔPHQ 2021.

### Statistical analyses

#### Data preprocessing

Data cleaning and analyses were performed in R v4.0.0 (R Core Team, 2020). The code used for preprocessing and analyses is available at https://osf.io/znwjt/.

Missing values (4.5%) were imputed by means of the MICE R package (Buuren & Groothuis-Oudshoorn, 2011) using classification and regression trees with *m* = 5 imputations and 50 iterations. Statistical outliers were all within the range of the used scales, therefore considered meaningful and not removed. Predictor variables were *z*-standardized; outcome variables were not *z*-standardized. This enabled (a) comparison between different PD outcomes irrespective of their variance and (b) clinically interpretable evaluation of the relation between psychological factors and absolute change in PD.

As shown in Table 1 and Fig. 1, pre-pandemic PD was calculated by averaging PHQ-4 scores from 2016 and 2019 to create a more robust baseline. A change in PD was then calculated by taking difference scores between pre-pandemic PD to 2020 (ΔPHQ 2020) and pre-pandemic PD to 2021 (ΔPHQ 2021). To better understand whether the examined psychological factors predicted change specifically during the COVID-19 pandemic or were generally related to changes in PD over time, we additionally investigated the relation of the predictors with the change in PD from 2016 to 2019 (ΔPHQ 2019).

#### Descriptive statistics

We conducted survey-weighted linear models to compare levels in PD between pre- and peri-pandemic survey waves.

#### Testing of main hypotheses

The above-mentioned hypotheses were tested using separate multiple linear regression analyses for each psychological factor/outcome pair, including all covariates in each model. This resulted in 10 regressions per outcome. Results were Bonferroni-corrected and hence considered significant at *p* < 0.005. Baseline PD levels were added to the models as an additional covariate to control for regression to the mean; we however refrain from reporting their associations with changes in PD. To counteract possible biases in sample selection and due to selective response rates, population survey weights were used (Kroh, 2009; Siegers, Steinhauer, & Dührsen, 2021). Because the survey weight was zero for 27 participants, final sample size was *n* = 6657 (*n* = 5981 at follow-up).

In order to determine which of the significant predictors found were most strongly associated with the outcomes in the multivariate setting with partly correlated variables, and at the same time avoid overfitting in a model with many predictors, we subsequently conducted LASSO (least absolute shrinkage and selection operator)-regularized regression analyses (Hastie, Tibshirani, & Wainwright, 2015) using the miselect R package (Rix & Du, 2020) and calculated inclusion frequencies. Details on this analysis can be found in online Supplement S3. Note that it was not possible to include survey weights into the LASSO analysis. As background information for interpretation of the unweighted LASSO results, a comparison of results from the unweighted linear regressions and weighted linear regressions can therefore be found in online Supplementary Table S3.

#### Additional analyses

As robustness analyses, we ran multiverse or specification curve analyses (Simonsohn, Simmons, & Nelson, 2020; Steegen, Tuerlinckx, Gelman, & Vanpaemel, 2016). Here, slightly different model specifications (linear *v.* robust regression, cube-root-transformation of non-normally distributed variables *v.* no transformation) were used to ensure that small arbitrary changes did not have major influences on the results of the study (see online Supplement S4).

To investigate how the psychological factors are associated with PHQ-4 in the individual years (*vs.* the change between years), we ran linear mixed models and estimated margins (mean ± 1 s.d.) for all predictors (see online Supplement S5).

## Results

### Sample description

**Fig. 2.**
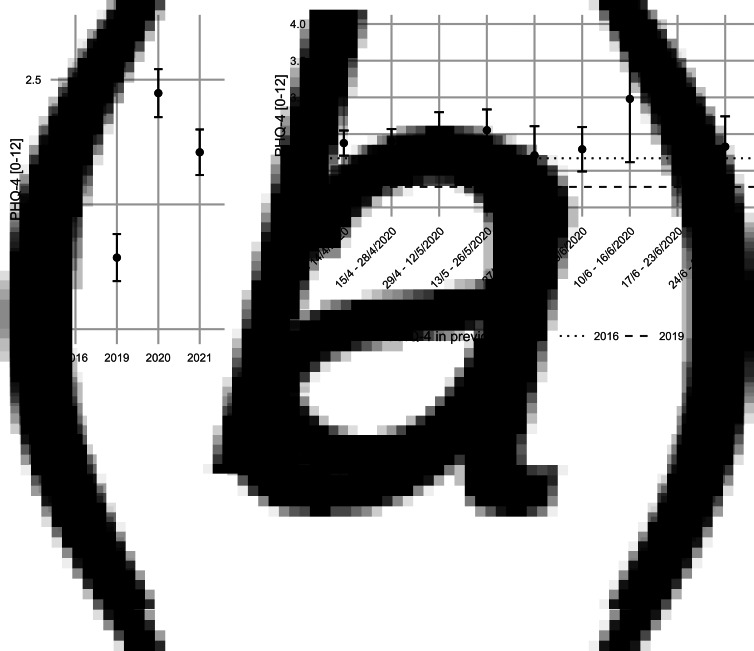
Psychological distress (PHQ-4) across years (*a*) and across the nine tranches ranging from 1 April to 28 June 2020 (*b*). *Note.* Error bars depict the 95% confidence interval. PHQ-4 values range from 0 to 12, higher values indicating higher PD. As weighted means are used, means of each individual tranche are representative for the German population. In (*b*), weighted mean PHQ-4 values of the entire sample in 2016 and 2019 are displayed as dotted and dashed horizontal lines, respectively.

Between April and June 2020, 31% (*v.* 20% in 2019 and 28% in 2016) of the population reported mild, 5% (*v.* 4% in 2019 and 6% in 2016) moderate, and 2% (*v.* 2% in 2019 and 2% in 2016) severe PD. Peri-pandemic PHQ-4 in 2020 (weighted *M* = 2.45/12, s.e.m. = 0.049) was significantly elevated compared to pre-pandemic levels in 2019 [weighted *M* = 1.79/12, s.e.m. = 0.048; *t*(6655) = 9.73, *p* < 2.2 × 10^−16^] and 2016 [weighted *M* = 2.17/12, s.e.m. = 0.061; *t*(6655) = 3.34, *p* = 0.002]. In January and February 2021, 29% reported mild, 5% moderate, and 2% severe symptoms. Peri-pandemic PHQ-4 in 2021 (*M* = 2.21/12, s.e.m. = 0.048) was significantly elevated compared to 2019 [*t*(6655) = 6.07, *p* = 1.345 × 10^−8^], but not compared to 2016 [*t*(6655) = 0.455, *p* = 0.653], and significantly lower than in 2020 [*t*(6655) = −3.41, *p* = 7.31 × 10^−4^]. Figure 2 displays weighted means and 95% confidence interval of the mean for PHQ-4 across the different years (panel *a*) and the nine individual tranches assessed in 2020 (panel *b*) Sample characteristics are displayed in Table 2.

**Table 2.** Sample characteristics (*N* = 6684)

	%
Gender	
Male	39.32
Female	60.68
Age	
18–24	2.5
25–34	9.38
35–44	16.37
45–54	23.21
55–64	21.27
65–74	15.61
75–84	9.63
85+	2.02
Education	
No degree, still in school, or lower degree	24.29
Middle or high school degree	43.88
High school degree with subsequent vocational training or university degree	31.83
Income	
Lower tertile	32.96
Medium tertile	33.49
Higher tertile	33.55
Risk group status for severe course in case of infection with SARS-CoV-2	
Yes	51.26
No	48.74
History of depression	
Yes	9.78
No	90.22

### Socio-demographic variables

With respect to socio-demographic factors, history of depression was positively related to ΔPHQ 2020 (*β* = 0.697), ΔPHQ 2021 (*β* = 1.063), and ΔPHQ 2019 (*β* = 2.060). Age group 18–24 (*β* = 1.075) and female gender (*β* = 0.419) were positively related to ΔPHQ 2021. All other socio-demographic variables were not significantly related to the outcomes. Exact relations of all covariates with the outcomes can be found in online Supplementary Tables S4–S6.

### Multiple linear regressions

As hypothesized, perceived recovery (*β* = −0.473) and reappraisal (*β* = −0.192) were negatively, whereas catastrophizing (*β* = 0.553) and neuroticism (*β* = 0.214) were positively related to ΔPHQ 2020. Contrary to our hypotheses, instrumental support-seeking (*β* = 0.282) was also positively related. All other predictors were not significantly associated with ΔPHQ 2020 (see online Supplementary Table S4).

As expected, perceived recovery (*β* = −0.332) and optimism (*β* = −0.139) were negatively, whereas catastrophizing (*β* = 0.259) and neuroticism (*β* = 0.355) were positively associated with ΔPHQ 2021. Instrumental support-seeking (*β* = 0.170) was again positively related. All other predictors were not significantly associated with ΔPHQ 2021 (see online Supplementary Table S5).

**Fig. 3.**
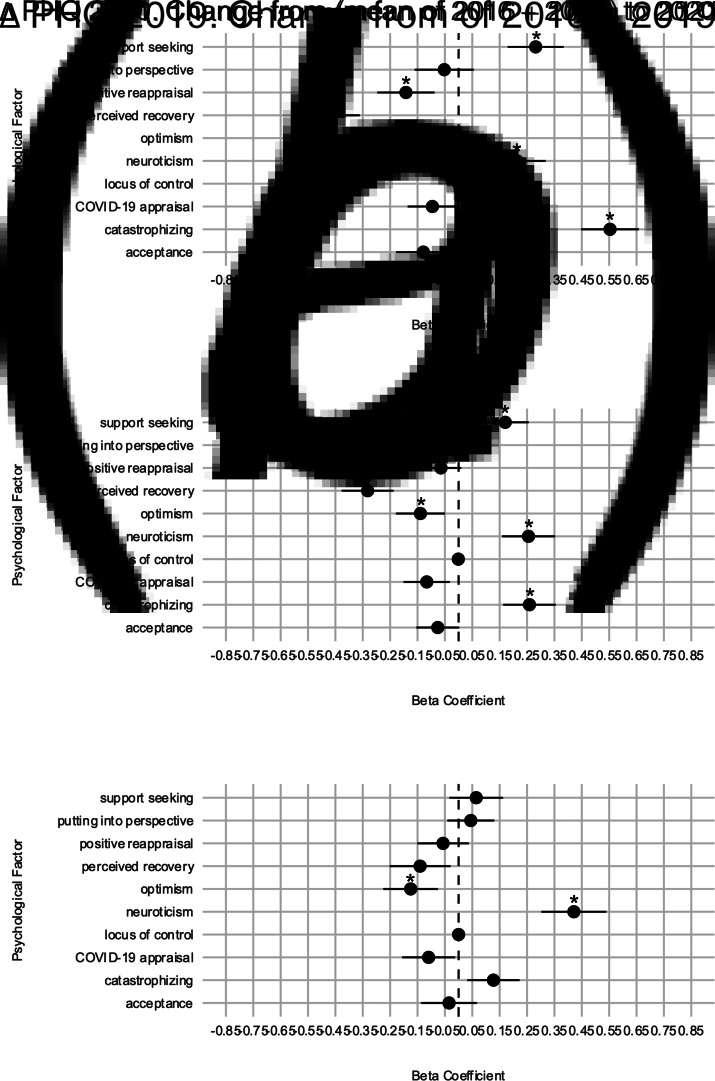
Beta coefficients of multiple linear regressions for ΔPHQ 2020 (*a*), ΔPHQ 2021 (*b*), and ΔPHQ 2019 (*c*). *Note*. This figure shows beta coefficients of the psychological factors for the three outcomes. Complete output tables of the respective linear regressions can be found in online Supplementary Tables S4–S6. Predictors are *z*-standardized, outcomes are not standardized. Error bars depict the 95% confidence interval.

To see if these factors were specifically relevant during the COVID-19 pandemic or also relevant before, we repeated the analyses with the change in PD during a control period (2016–2019) as the outcome (see online Supplementary Table S6). Optimism (*β* = −0.175) was negatively, whereas neuroticism (*β* = 0.421) was positively associated with ΔPHQ 2019. All other psychological factors were not related. Beta coefficients for all psychological factors and all outcomes are shown in Fig. 3.

### LASSO-regularized regressions

LASSO-regularized regression analysis highlighted the roles of catastrophizing, perceived recovery, neuroticism, and asking for instrumental support for ΔPHQ 2020, of neuroticism, perceived recovery, and catastrophizing for ΔPHQ 2021, and of neuroticism as well as optimism for ΔPHQ 2019 (see online Supplementary Table S7).

### Specification curve analyses

The performed specification curve analyses indicate that results remained stable across model specifications (see online Supplement S4).

### Linear mixed models

Linear mixed models revealed similar patterns of predictors for pre- *v.* peri-pandemic PHQ-4 compared to the multiple linear regressions on ΔPHQ outcomes (see online Supplement S5).

## Discussion

The goal of this study was to investigate if mean PD increased compared to pre-pandemic times and which risk and resilience factors are associated with the change in PD in a sample representative of the German household population.

First, as expected, we found that PD was on average significantly higher in both 2020 and 2021 compared to 2019. It however must be mentioned that pre-pandemic PD in 2019 was lower than in 2016. Due to these PD fluctuations at baseline, averaged baseline scores were used in all subsequent analyses.

Second, in line with our hypotheses, we found catastrophizing and neuroticism to be risk factors for PD. Unexpectedly, asking for instrumental support also was positively associated with PD across peri-pandemic outcomes.

Third, the most consistent protective factor across all analyses was self-perceived recovery from stress, whereas other factors like optimism and positive reappraisal where only partially supported as protective factors. Contrary to our expectations, putting things into perspective, acceptance, and positive appraisal specific to the COVID-19 pandemic did not emerge as protective factors. We will discuss these results in more detail below.

### PD increase in the general population

In our analyses there was an increase in PD in 2020 (2.45) and 2021 (2.21) compared to 2019 (1.79). PD in 2020, but not 2021, was also higher than in 2016 (2.17). PD in 2021 was again significantly lower than that in 2020.

These average numbers are clearly not in the pathological range, as scores of 6 and higher reflect moderate to severe PD. However, systematic increases of average PD into the pathological range can hardly be expected in a sample consisting of over 6000 participants. Especially, the proportion of participants reporting mild (*v.* no) symptoms was elevated compared to pre-pandemic times. Our findings of only small but significant increases during the pandemic are in accordance with many other findings in population-based studies (Daly et al., 2020; Niedzwiedz et al., 2020; Peters et al., 2020; Pierce et al., 2020; Twenge & Joiner, 2020). Intriguingly, this small effect may be caused by (vulnerable) subpopulations as indicated by longitudinal samples (e.g. Ahrens et al., 2021).

Existing meta-analytic evidence suggests a recovery of PD over time (Robinson et al., 2021). In our sample, PD in 2021 was still elevated, which we attribute to the fact that unlike the studies included in the meta-analysis, we covered a later time point in the middle of another wave of COVID-19 infections. PD in 2021 was however lower than that in 2020. This might on the one hand be explained by a habituation effect to the pandemic consequences, including an adjustment to the changes in daily life and social distancing measures. On the other hand, the existence of more precise knowledge about the virus and the prospect of starting vaccination campaigns in Germany in the beginning of 2021 might have led to lower uncertainty compared to 2020 and therefore a different appraisal of the situation, which in turn differentially influenced mental well-being.

### Risk factors for PD

Female gender and younger age were socio-demographic risk factors for peri-pandemic PD in 2021 but not in 2020, adding to the mixed picture that although many studies reported these to be risk factors (see Introduction), meta-analytic evidence did not find this relationship (Robinson et al., 2021). The most important psychological risk factor was catastrophizing as it showed positive associations with PD changes across peri-pandemic analyses (but not in the control analyses for the pre-pandemic change from 2016 to 2019). Catastrophizing is the tendency to think that things are worse than they are or will have a far worse outcome than is realistic. Confirming previous research that highlights catastrophizing as one of the most prominent emotion regulation strategies predicting PD (Garnefski & Kraaij, 2007; Martin & Dahlen, 2005), our results indicate that this type of coping is the most maladaptive of those included as predictor. Neuroticism also showed a positive association with PD outcomes in almost all analyses, also for the pre-pandemic control analyses, an association that is well known from the literature (Lahey, 2009). Unexpectedly, asking for instrumental support as coping strategy also emerged as quite consistently positively associated with PD, contrary to what we hypothesized. However, it is conceivable that this predictor was confounded with having negative experiences or symptoms in the first place. The specific formulation of this item was: ‘I've been trying to get advice or help from other people about what to do’. People might have only reached out to other people for help if they already experienced significant burden, whereas individuals with less burden might not have sought to do so, especially under the given pandemic circumstances.

### Protective factors for PD

Overall, we found perceived recovery from stress to be the most consistent protective factor across peri-pandemic analyses. Optimism and positive reappraisal were at least partially found to be protective factors, consistent with previous research on their association with mental health (Martin & Dahlen, 2005; Plomin et al., 1992). We did not find support for putting things into perspective, acceptance, and positive appraisal specific to COVID-19 to be protective factors.

Optimism and perceived recovery were the only protective factors associated also with PD change in the pre-pandemic control period.

These findings could thus indicate that the results regarding other psychological protective factors such as positive reappraisal are specific to the COVID-19 pandemic and that they are not related to changes in PD under normal circumstances. However, an additional, and more likely, explanation is that the temporal distance between the assessment of the pre-pandemic PD change score on the one hand and psychological factors assessed in 2020 on the other hand is too large to find associations. The fact that coping items such as positive reappraisal were reformulated to reflect state-like coping in 2020 substantiates this possible explanation, especially since perceived stress recovery, a trait-like measure assessed in the same wave, does show relation to ΔPHQ 2019.

### Limitations

Despite the strengths of our study such as the representativeness of the sample and existence of individual pre-pandemic baseline PD, which have been considered important specifically in the context of the COVID-19 pandemic (Kunzler et al., 2021; Nieto, Navas, & Vázquez, 2020), as well as the comparison with change in PD during a pre-pandemic period and the use of LASSO-regularized regression that selects the most promising variables in a model with many potential variables, there also are several limitations: most importantly, psychological factors were not assessed at all survey waves (see Fig. 1). Given that many psychological factors such as coping may be variable and malleable (Compas, Forsythe, & Wagner, 1988), this impedes disentangling directionality of causation between psychological factors, PD, and pandemic context. We are also aware of the second major limitation that results from the uneven sampling of psychological factors: we were forced to include variables that were assessed in 2020 to the model predicting change from 2016 to 2019. Our rationale to nevertheless include the factors into the model was to keep the models as similar as possible to set the peri-pandemic results into perspective. We moreover included variables that were assessed during previous survey waves, such as locus of control in 2015, neuroticism in 2017, and optimism in 2019. Although it would have been preferred to have more recent data, based on the literature we expect a relative stability of these constructs (Cobb-Clark & Schurer, 2011, 2013; Scheier, Carver, & Bridges, 1994). Other limitations are the self-report nature of assessments and that the use of single items instead of entire validated questionnaires to assess the coping dimensions, although necessary for pragmatic reasons, might have led to a reduced statistical power. Finally, we do not have knowledge of specific stressors that might have occurred between the two measurements; changes in PD from baseline to 2020 and baseline to 2021 can therefore only be partly, and only on average, attributed to experiencing the COVID-19 pandemic. However, although there undoubtedly are other influences on changes in PD that we did not assess and that therefore cannot be controlled for, these are expected to occur at random, whereas every participant experienced the COVID-19 pandemic during data collection.

### Outlook

In the present research, we identified several psychological factors that are associated with changes in PD during the COVID-19 pandemic in the general population of Germany. Although due to the used instruments our results can strictly only give insights regarding PD, in light of past findings (Veer et al., 2021) we also expect these psychological factors to be related to general mental health and resilience (i.e. mental health controlled for stressor exposure; see Kalisch et al., 2021 for further details). The exact pattern of predictors might certainly be different when investigating these slightly different outcomes. For example, it should be noted that the strongest predictors for PD in our study were those that are conceptually closest to PD (catastrophizing, asking for instrumental support, neuroticism, and perceived stress recovery), whereas psychological factors that are conceptually further away from symptoms such as positive reappraisal, optimism, or locus of control, show weaker relationships with PD. These latter factors however seem to be stronger predictors for resilience, as for instance shown in Veer et al. (2021). Future representative studies should investigate this in more detail.

Our results do point to some possibly malleable factors that we found to be prospectively associated with changes in PD during COVID-19 and that are therefore possible candidates for targeted prevention and intervention programs to improve general mental well-being during challenging times such as pandemics. Given the pandemic situation, these prevention efforts should ideally be widely accessible and allow for a remote delivery via internet and/or mobile phone. Above all, improving stress recovery, e.g. via physical exercise in the nature (Wooller, Rogerson, Barton, Micklewright, & Gladwell, 2018) or smartphone-assisted biofeedback (Hunter, Olah, Williams, Parks, & Pressman, 2019), appears to be the most promising starting point. Moreover, reducing catastrophizing tendencies, for example via smartphone-based cognitive behavioral interventions for mental health prevention (Ebert et al., 2018; Marciniak et al., 2020), increasing internal locus of control, e.g. via online interventions as has been done by Nallapothula et al. (2020) in an academic context, increasing optimism, e.g. using the best possible self intervention (Malouff & Schutte, 2017), and learning to also see positive aspects in the overall challenging situation, e.g. via mobile cognitive behavioral interventions (Ebert et al., 2018; Marciniak et al., 2020) are promising paths to increase individual well-being. Future research should corroborate these directions using interventional studies.

## Supplementary material

For supplementary material accompanying this paper visit https://doi.org/10.1017/S0033291722000563. click here to view supplementary material
