# Supplementary material for: Coping with COVID: risk and resilience factors for mental health in a German representative panel study
Source: Psychol Med. 2022 Mar 1;53(9):3897–907. doi: 10.1017/S0033291722000563 (PMC8943230; doi:10.1017/S0033291722000563)

**Supplementary Materials**

*Table S1.* COVID-related keywords

| COVID keywords | English translation |
| --- | --- |
| 肺炎 | pneumonia |
| (新)冠(狀/状)(病毒) | coronavirus |
| covid/COVID | covid/COVID |
| 疫情 | epidemic/pandemic |
| 武漢(汉) | Wu Han |
| (鑽/钻)石公主(號号)(郵輪/邮轮) | Diamond Princess  Cruise ship |

*Table S2*. Variables of interest used in time series analyses.

| Variables | Description |
| --- | --- |
| Open Up | Mentioning COVID-19 in Open Up |
| CMD | Mentioning COVID-19 and common mental disorder (CMD) |
| NO CMD | Mentioning COVID-19 without common mental disorder |
| COVID-19 | Number of COVID-19 confirmed case in Hong Kong (Daily/Weekly) |
| 1st Wave | Wave of COVID-19 pandemic from January 24 to February 14 |
| 2nd Wave | Wave of COVID-19 pandemic from March 17 to April 11 |
| 3rd Wave | Wave of COVID-19 pandemic from July 1 to August 30 |
| 4th Wave | Wave of COVID-19 pandemic from November 20, 2020 to January 28, 2021 |
| ImpF-1 | impulse function: 1st wave of COVID-19 = 1; rest = 0 |
| ImpF-2 | impulse function: 2nd wave of COVID-19 = 1; rest = 0 |
| ImpF-3 | impulse function: 3rd wave of COVID-19 = 1; rest = 0 |
| ImpF-4 | impulse function: 4th wave of COVID-19 = 1; rest = 0 |
| MagI-1 | magnitude effect: COVID-19 magnitude in the 1st wave |
| MagI-2 | magnitude effect: COVID-19 magnitude in the 2nd wave |
| MagI-3 | magnitude effect: COVID-19 magnitude in the 3rd wave |
| MagI-4 | magnitude effect: COVID-19 magnitude in the 4th wave |

*Figure S1*. Trends of different CMD mentioned sessions from January 2019 to January 2021.


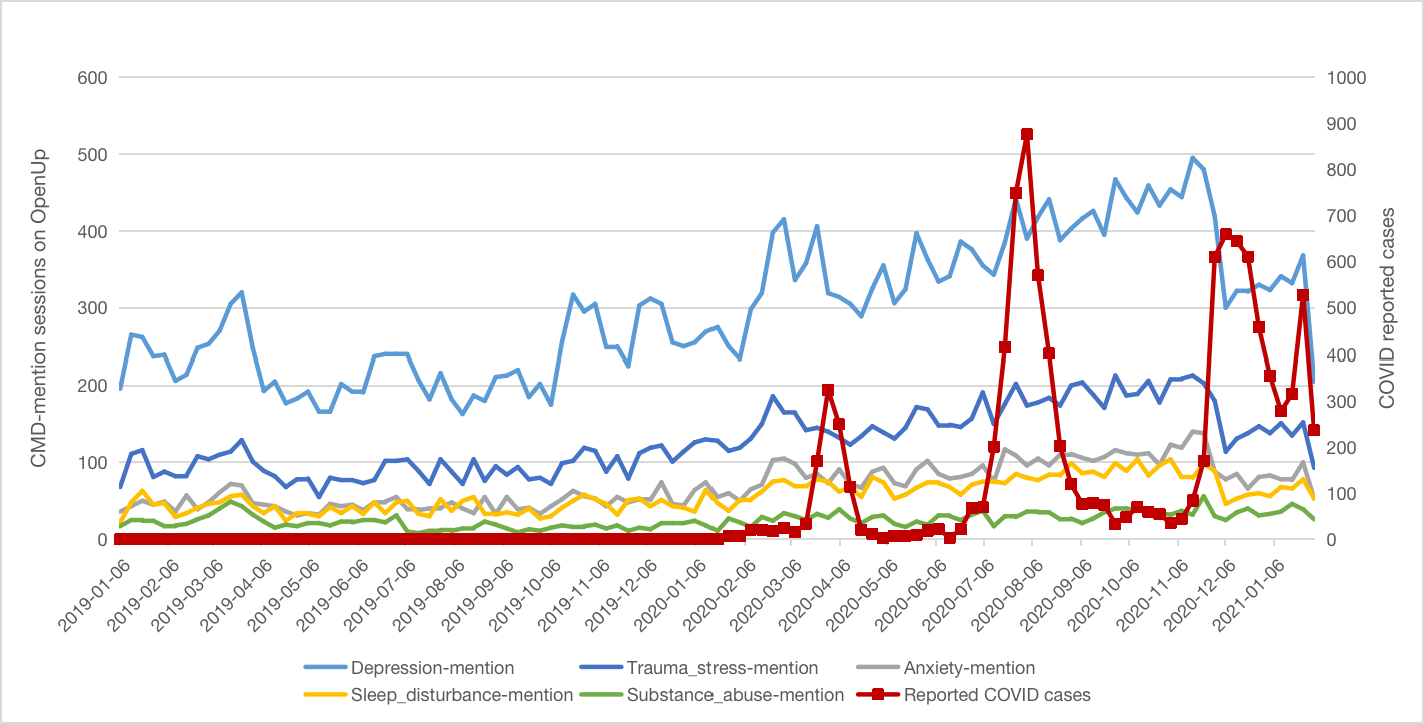

Supplement: Supplementary file 1 [file S0033291722000563sup001.docx]
